# Do radioiodine-avid lymph nodes from differentiated thyroid cancer on the initial posttherapy scan need repeated ^131^I therapy?

**DOI:** 10.3389/fendo.2023.1099449

**Published:** 2023-05-30

**Authors:** Yongji Jiang, Simin Liu, Xiaotong Qiu, Yanlei Huo, Xiaoying Zhang, Haidong Cai, Zhongwei Lv, Chao Ma

**Affiliations:** Department of Nuclear Medicine, Shanghai Tenth People’s Hospital, School of Medicine, Tongji University, Shanghai, China

**Keywords:** differentiated thyroid cancer (DTC), ^131^I therapy, lymph node metastasis, treatment response, posttherapy scan

## Abstract

**Background:**

Residual/recurrent lymph node metastase (LNM) is often found after differentiated thyroid cancer (DTC) surgery. This study aimed to investigate whether patients complicated with radioiodine-avid (^131^I+) lymph nodes from DTC on the initial posttherapy scan (PTS) need repeated ^131^I therapy.

**Methods:**

From June 2013 to August 2022, DTC patients with ^131^I+ lymph nodes on the initial PTS who received at least two cycles of ^131^I therapy were retrospectively enrolled. They were divided into a complete response (CR) group and an incomplete response (IR) group according to their response to the initial ^131^I therapy based on the 2015 American Thyroid Association (ATA) guidelines.

**Results:**

A total of 170 DTC patients with ^131^I+ lymph nodes on the initial PTS were included; 42/170 (24.7%) patients were classified into the CR group and 128/170 (75.9%) were classified into the IR group according to their response to the initial ^131^I therapy. None of the 42 CR patients had disease progression at the subsequent follow-up, and 37/170 (21.8%) IR patients improved after repeated therapy. Univariate analysis showed that N stage (*P=*0.002), stimulated thyroglobulin (sTg) level before initial ^131^I therapy (*P*<0.001), LNM size (*P*<0.001), number of total residual/recurrent LNM (*P=*0.021), radioiodine-nonavid (^131^I-) LNM (*P=*0.002) and ultrasound features (*P*<0.001) were related to the initial treatment response. On multivariate analysis, sTg level (*OR*=1.186, *P*<0.001) and LNM size (*OR*=1.533, *P*=0.004) were independent risk factors for IR after initial ^131^I therapy. The optimal sTg level and LNM size cutoff value for predicting the treatment response after initial ^131^I therapy were 18.2 µg/l and 5mm.

**Conclusion:**

This study suggested that approximately one-quarter of patients with ^131^I+ lymph nodes on initial PTS, especially those with N0 or N1a stage, lower sTg level, smaller LNM size, ≤2 residual/recurrent LNMs, negative ultrasound features and no ^131^I- LNM, remain stable after one cycle of ^131^I therapy and do not need repeated therapy.

## Introduction

Differentiated thyroid cancer (DTC) is the most common type of thyroid cancer, and the incidence rate of DTC has increased in recent decades (1). The lymph node is the most common site of DTC metastasis, and approximately 30-80% of patients have lymph node metastase (LNM) (2). Furthermore, residual/recurrent LNM can often be found in many postoperative DTC patients at the time of initial ^131^I therapy. Therefore, proper treatment methods are necessary to remitlocal symptoms and psychological stress. ^131^I therapy is an excellent choice for patients with lymph nodes with iodine uptake capacity (3). However, the therapeutic efficacy of ^131^I for DTC patients with ^131^I+ lymph nodes on the initial posttherapy scan (PTS) has not been reported. Thus, determining which patients may or may not benefit from repeated ^131^I therapy is challenging. Consequently, we analyzed the factors influencing the treatment response after initial ^131^I therapy and obtained the clinical outcomes of these patients.

## Subjects and methods

### Patients

A retrospective analysis was performed of the clinicopathological data of postoperative DTC patients with ^131^I+ LNM on initial PTS from June 2013 to August 2022 in our department (Department of Nuclear Medicine, Shanghai Tenth People’s Hospital). The inclusion criteria were as follows: (a) total thyroidectomy and pathological type was papillary or follicular thyroid cancer (PTC or FTC), and (b) the initial PTS showed residual/recurrent ^131^I+ LNM and at least had two PTSs (in order to prevent the diagnostic scan was false negative). The exclusion criteria were as follows: (a) complicated with distant metastases; (b) follow-up time less than 6 months; and (c) operation or other treatment after ^131^I therapy.

### Therapeutic approach and follow-up strategy

Before ^131^I therapy, L-thyroxine (L-T_4_) treatment was discontinued for 3-4 weeks until serum thyroid stimulating hormone (TSH) levels increased above 30 mIU/l. All patients were routinely examined for TSH, free triiodothyronine (FT_3_), free thyroxine (FT_4_), stimulated thyroglobulin (sTg), and Tg antibody (TgAb, <115 IU/ml was considered to be negative) levels and underwent neck ultrasound. Doses of ^131^I ranging from 3.70-5.55 GBq (100-150 mCi) were delivered based on the 2015 American Thyroid Association (ATA) risk classification system (3) and were properly increased or decreased according to age or individual status. All included patients underwent at least two cycles of ^131^I therapy, and the second dose (119.7 ± 41.2 mCi) was similar to the initial therapeutic dose (122.8 ± 32.2 mCi). Patients were discharged with a TSH-suppressive dose of L-T_4_, 2.0-2.5 μg/kg, after ^131^I therapy. The treatment cycle of ^131^I at our hospital is approximately 6 months. All patients received regular follow-up for TSH, Tg, TgAb, and neck ultrasound assessment for 6-104 months (median time: 13 months).

### Diagnostic criteria for lymph node metastases

The diagnosis of LNM from DTC was established based on the serum Tg and TgAb levels, neck ultrasound and CT before or after ^131^I therapy, and ^131^I avidity on PTS. LNM from DTC was confirmed by the following criteria: (a) pathological puncture results; (b) positive ^131^I accumulation of lymph node based on PTS; (c) ultrasound features of LNM including absence of a hilum, round shape, increased short axis, increased central vascularization, cystic area, microcalcification, and peripheral or diffusely increased vascularization (4); and (d) unenhanced center and rim enhancement, with or without fine sand calcification and mottle calcification on CT or lymph node with spherical shape and minimal axial diameter >10 mm and an increase in size on follow−up CT (5). N stage matched the eighth edition of the American Joint Committee on Cancer (AJCC)/TNM staging system for thyroid cancer (6).

### Posttherapy scan

The PTS was acquired within 2-4 days after oral administration of ^131^I, and it was obtained in the anterior and posterior projection with a large field-of-view gamma camera equipped with a high-energy collimator (360 keV, 20% energy window). All patients underwent neck and superior mediastinum fusion SPECT/CT to locate ^131^I+ lesions. Patients with ^131^I+ LNM can be considered positive for PTS.

### Treatment response classification

Treatment responses were classified into 4 types according to the 2015 ATA guidelines (3): excellent response (ER), indeterminate response (IDR), biochemical incomplete response (BIR), and structural incomplete response (SIR). In our study, the four responses were divided into CR (complete response, including ER) and IR (incomplete response, including IDR, BIR, and SIR) according to the initial therapy response. The initial therapy response was evaluated at the time of the second therapy cycle based on the second PTS, Tg and ultrasound (6 months after the initial therapy).

### Statistical analysis

Continuous data are expressed as the mean ± standard deviation; categorical data are expressed as frequencies and percentages. Categorical comparisons were performed with the chi-square test, and continuous data were compared using a two-sample *t* test and the *Mann–Whitney U* test. Logistic regression analysis was used for the multivariate analysis of independent variables that had *P* values of <0.05 in the univariate analysis. Receiver operating characteristic (ROC) curves were applied to evaluate the value of sTg (Negative TgAb) level and LNM size for predicting the initial ^131^I treatment response. All tests were two sided, and a *P* value <0.05 was considered statistically significant. Statistical analyses were performed using SPSS version 26.0.

## Results

**Figure 1:**
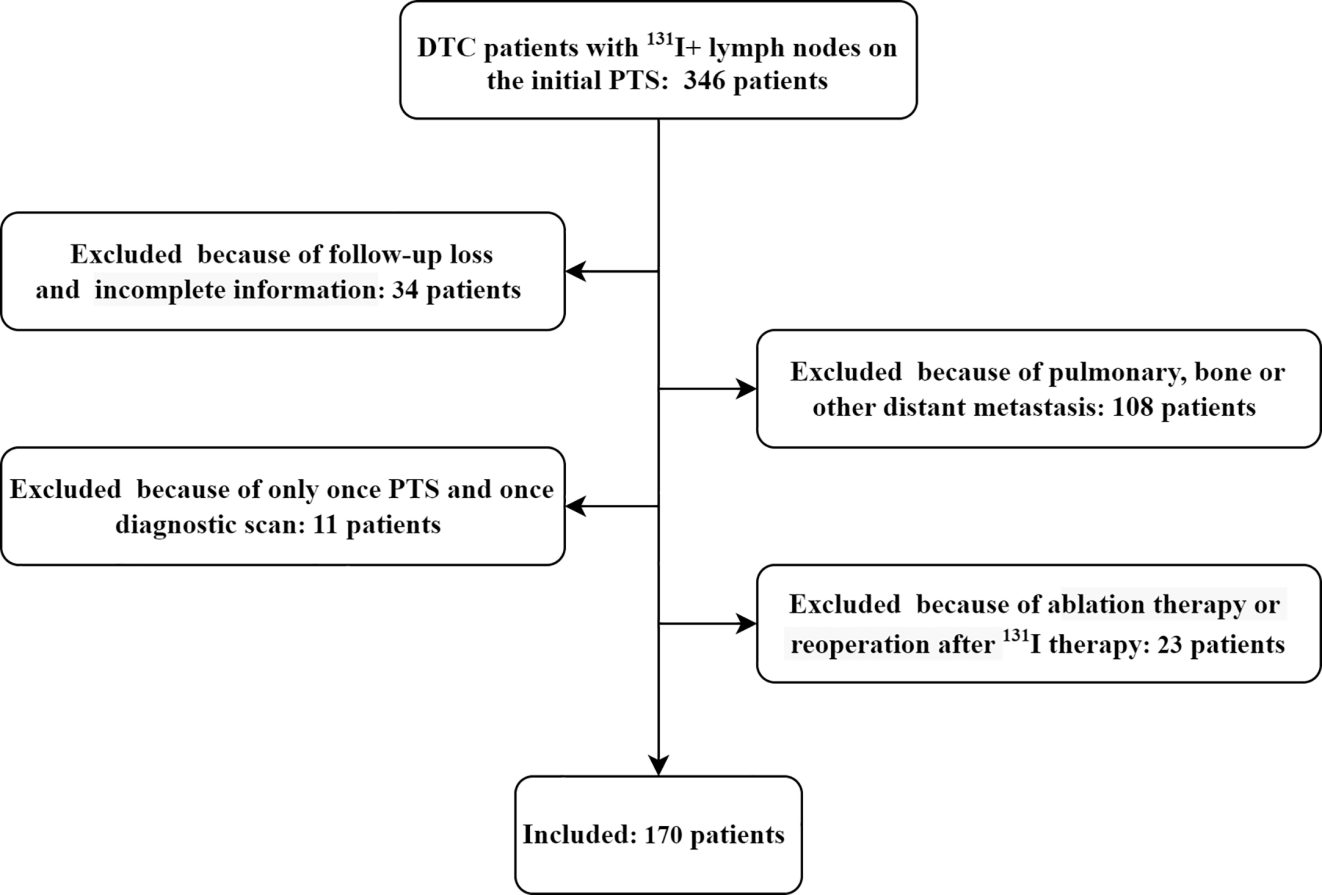
Flow chart of enrollment of study patients.

**Figure 2:**
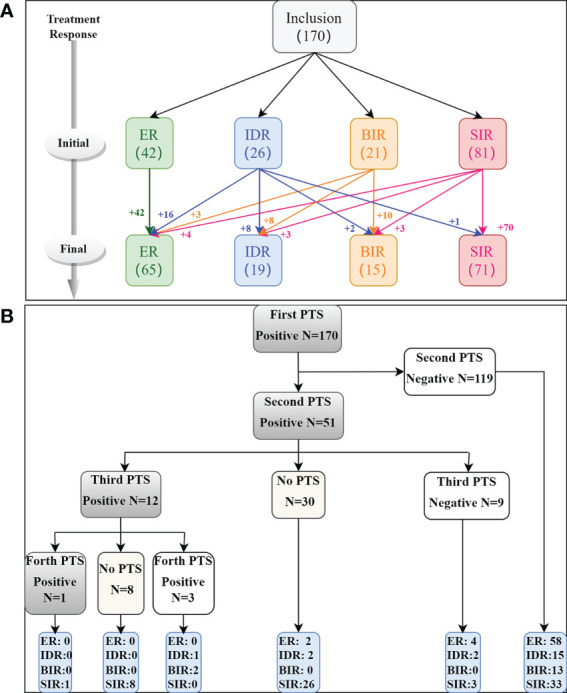
Flow chart of treatment response **(A)** and PTS **(B)** of DTC patients after 131I therapy.

A total of 170 DTC patients were included in the study (**Figure 1**); 75 patients were male, and 95 were female, and the average diagnostic patient age was 44.2 ± 13.6 years (range 14-75 years). A total of 295 lymph nodes were identified as LNMs, including 248 ^131^I+ LNMs and 47 ^131^I- LNMs. All patients underwent two cycles of therapy, and 21 and 4 patients underwent three and four cycles of therapy, respectively. CR was achieved in 42/170 (24.7%) patients after the initial ^131^I therapy, and IR was achieved in 128/170 (75.3%) patients (including 26 IDR, 21 BIR, 81 SIR). None of the ER patients progressed to IR at follow-up, and the disease remained stable. At the end of follow-up, 37 IR patients (16 IDR, 11 BIR, 10 SIR) improved after repeated ^131^I therapy, 65/170 (38.2%) were classified as CR, and 105/158 (61.8%) were classified as IR (including 19 IDR, 15 BIR, 71 SIR) (**Figure 2A**). A total of 119/170 (70.0%) patients’ second PTS was negative, and 51/170 (30.0%) had a positive second PTS. Patients with a negative second PTS did not undergo a third cycle of therapy. Of the 51 patients with a positive second PTS, 21/51 underwent three cycles of therapy, while 30/51 did not undergo further ^131^I therapy. Four patients underwent four cycles of therapy, and only one patient’s fourth PTS was positive (**Figure 2B**).

**Figure 3:**
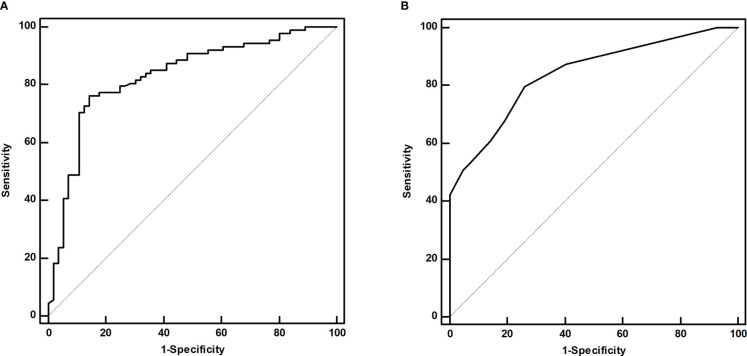
The ROC curves of the sTg level **(A)** and LNM size **(B)** for predicting the initial treatment response.

The univariate analysis showed that CR after initial ^131^I therapy tended to be observed in patients with N0 or N1a stage (*χ^2 = ^* 9.303, *P*=0.002), lower sTg level (*Z*=-6.707, *P*<0.001), smaller LNM size (*Z*=-6.665, *P*<0.001), ≤2 LNMs (*χ^2 = ^* 5.367, *P*=0.021), no ^131^I- LNM (*χ^2 = ^* 9.870, *P*=0.002), and negative ultrasound features (*χ^2 = ^* 22.200, *P*<0.001). Furthermore, the initial ^131^I doses (*Z*=-3.214, *P*<0.001) in patients with IR were higher than those in patients with CR with poor clinical pathology (**Table 1**). Multivariate logistic regression showed that the sTg level (odds ratio[*OR*]=1.186, 95% *CI*: 1.072-1.313, *P*<0.001) and LNM size (*OR*=1.533, 95% *CI*: 1.149-2.045, *P*=0.004) were independent risk factors associated with the initial therapy response (**Table 2**). The area under the ROC curve (AUC) was 0.831 (95% *CI*: 0.760-0.888), and the best sTg cutoff point for predicting the initial treatment response was 18.2 µg/L; at this level, the sensitivity and specificity were 76.1% and 85.7%, respectively (**Figure 3A**). The AUC was 0.841 (95% *CI*: 0.777-0.892), and the best LNM size cutoff point for predicting the initial treatment response was 5mm; at this size, the sensitivity and specificity were 79.7% and 73.8%, respectively (**Figure 3B**).

**Table 1:** Clinicopathological characteristics compared based on the initial treatment response.

	CR	IR	*P* value
Number of patients	42	128	
Age at diagnosis(year)	46.4 ± 9.4	43.5 ± 14.7	0.234^a^
Gender			0.214^b^
Male	22(52.4)	53(41.4)	
Female	20(47.6)	75(58.6)	
Tumor types			1.000^b^
Papillary	41(97.6)	126(98.4)	
Follicular	1(2.4)	2(1.6)	
Multifocal cancer			0.535^b^
Yes	20(47.6)	60 (46.9)	
No	22(52.4)	68(53.1)	
Tumor size(cm)	1.4(0.8-2.1)	1.5(1.0-2.0)	0.598^d^
Capsule invasion			0.531^b^
Yes	26(61.9)	86(67.2)	
No	16(38.1)	42(32.8)	
N stage			**0.002** ^b^
N0+N1a	26(61.9)	45(35.2)	
N1b	16(38.1)	83(64.8)	
TgAb			0.776^b^
Negative	35(83.3)	109(85.2)	
Positive	7(16.7)	19(14.8)	
sTg level (μg/l)	5.1(1.5-9.5)	30.9(13.6-109.9)	**<0.001** ^d^
LNM size (mm)	4.0(3.0-6.0)	10.0(6.0-14.0)	**<0.001** ^d^
Number of ^131^I+ LNM			0.678^c^
≤2	39(92.9)	114(89.1)	
>2	3(7.1)	14(10.9)	
Number of total LNM			**0.021** ^b^
≤2	39(92.9)	98(80.6)	
>2	3(7.1)	30(19.4)	
^131^I^-^ LNM			**0.002** ^b^
Yes	1(2.4)	31(24.2)	
No	41(97.6)	97(75.8)	
Ultrasound feature			**<0.001** ^b^
Negative	34(81.0)	50(39.1)	
Positive	8(19.0)	78(60.9)	
Initial ^131^I dose (mCi)	100.0(100.0-120.0)	120.0(100.0-150.0)	**0.001** ^d^

CR, complete response (including ER); IR, incomplete response (including IDR, BIR and SIR); LNM, lymph node metastase; ^131^I+, ^131^I-avid; ^131^I-, ^131^I-nonavid; TgAb, Tg antibody; sTg (Negative TgAb), stimulated thyroglobulin; ^a^, t test; ^b^, Pearson Chi-squared test; ^c^, Chi-squared test (continuity correction); ^d^, Mann–Whitney U test; Bold value means P<0.05.

**Table 2:** Multivariate binary logistic regression of association factors for predicting the initial treatment response.

	Initial treatment response		
	*OR*	Hazard Ratio (95% *CI*)	*P* Value
N stage (N0+N1a/N1b)	2.857	0.815-10.014	0.101
sTg level (μg/l)	1.186	1.072-1.313	**<0.001**
Number of total LNM (≤2/>2)	1.199	0.135-10.672	0.871
^131^I- LNM (No/Yes)	0.399	0.011-14.590	0.617
LNM size (mm)	1.533	1.149-2.045	**0.004**
Ultrasound feature (Negative/Positive)	4.159	0.933-18.533	0.062

Bold value means P<0.05.

## Discussion

DTC is prone to lymph node metastasis, and LNM can often be found in intermediate- or high-risk patients after central or lateral neck dissection. The main reasons are incomplete nodal dissection and lack of preoperative identification (7). ^131^I therapy is an effective method for treating postoperative DTC patients with residual/recurrent LNM, especially LNM with good ^131^I uptake capacity, and the effective rate can reach more than 80% (7). Clinical work has shown that pulmonary and bone metastases maintain the ability of iodide uptake after repeated ^131^I therapy and have poor prognosis (8, 9). In the present study, we found that approximately 70.0% of DTC patients with ^131^I+ LNM on the initial PTS might  have negative results at the time of the second therapy cycle, which means that these patients have a good response to ^131^I therapy or become radioiodine-refractory. Therefore, not every DTC patient with ^131^I+ LNM needs repeated ^131^I therapy. However, no studies have reported and reviewed the features of patients in whom repeated ^131^I therapy is unnecessary.

In our present study, 24.7% (42/170) of patients were classified as ER after initial therapy, and their disease conditions remained stable at the subsequent follow-up. Therefore, it is necessary and meaningful to investigate the features of patients who do or do not need repeated ^131^I therapy. Yu (10) studied 206 intermediate- to high-risk DTC patients after ^131^I therapy and found that ER was achieved in 116/206 patients 6 months after initial therapy and remained stable after 12 months or even longer. Patients in our study also had intermediate- to high-risk DTC, which was consistent with his research. ER patients only have a 1-4% chance of recurrence (3). Therefore, if patients can be cured by one cycle of ^131^I therapy, the second ^131^I cycle may not have benefits and may cause other side effects. Comprehensive evaluation and individualized treatment can prevent a significant number of unnecessary therapy cycles. A total of 37/170 (21.8%, 1 IDR, 11 BIR, 10 SIR) patients improved after repeated ^131^I therapy. Although 17/38 patients were still classified as IR, their serological indicators and lesion size improved, which indicated that repeated therapy may be effective. The cure rate (38.2%) of ^131^I therapy for patients with ^131^I+ LNM in our study was lower than that in other studies related to LNM (11, 12). The reason may be due to the different inclusion and treatment response evaluation criteria.

**Figure 4:**
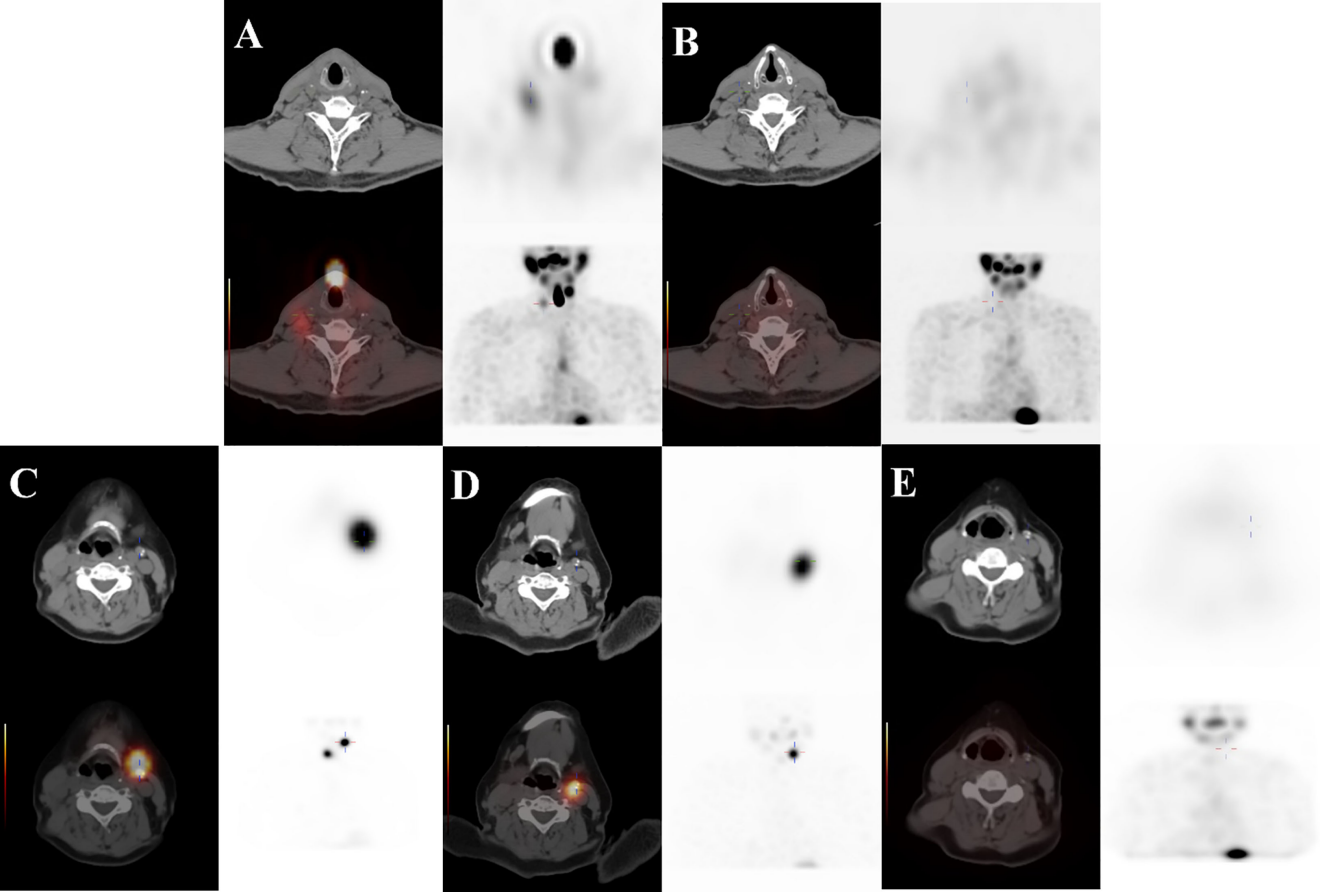
① A 71-year-old male DTC patient with ^131^I+ LNM on the right neck at Level III. **(A, B)** The first PTS was positive (TSH: 70.411 mIU/l, Tg: 19.2 μg/l, TgAb: <10 IU/ml), and the second PTS was negative (TSH: 82.545 mIU/l, Tg: 1.97 μg/l, TgAb: <10 IU/ml). She was classified as ER at the end of follow-up (TSH: 0.891 mIU/l, Tg: 0.04 μg/l, TgAb: <10 IU/ml, negative ultrasound features). ② A 67-year-old female DTC patient with ^131^I+ LNM on the left neck at Level II. **(C–E)** The patient’s first two PTSs were positive, and the third PTS was negative (TSH: 57.084 mIU/l, Tg: 39.58 μg/l, TgAb: <10 IU/ml→TSH: 88.895 mIU/l, Tg: 8.68 μg/l, TgAb: <10 IU/ml→TSH: 74.001 mIU/l, Tg: 3.79 μg/l, TgAb: <10 IU/ml), and she was classified as IDR at the end of follow-up (TSH: 0.019 mIU/l, Tg: 0.04 μg/l, TgAb: <10 IU/ml, calcification on ultrasound).

Our results showed that N stage, the number of residual/recurrent LNM, ^131^I- LNM, ultrasound features, LNM size and sTg level were related to the initial treatment response. Patients with N1b stage and multiple residual/recurrent LNMs (>2) were likely to undergo repeated therapy cycles and have poor treatment response (13). These results may be related to advanced invasion and poorly differentiated subtypes, and these patients are prone to have persistent disease and recurrent lesions. ^131^I- LNM, often found by ^18^F-FDG PET/CT and ultrasound, usually have poor differentiation and advanced malignant degrees and are insensitive to ^131^I therapy. Ultrasound plays an important role in evaluating therapeutic effects, and negative ultrasound features indicate that LNM are in an early stage and only have metabolic changes without morphological changes. Hence, CR tends to be achieved in patients with negative ultrasound findings. According to the 2015 ATA guidelines, SIR is defined as structural or functional evidence of disease with any Tg level. However, our study found that some ultrasound abnormal lymph nodes remain stable for a long time after ^131^I therapy with extremely low sTg levels (<0.04 μg/l, TgAb<10 IU/ml), and the therapeutic response is difficult to classify (might be ER or SIR, our study classified this condition as IDR) (**Figure 4**). Robenshtok (14) held that these lymph nodes were very unlikely to cause local complications or be associated with the development of distant metastases if followed carefully with serial ultrasound examinations. Based on this, we propose a hypothesis that partial LNMs with abnormal structures may be classified as ER due to the deactivation of tumor cells and only have abnormal morphological structures. However, there is no literature that can validatethis. Therefore, pathology, immunohistochemistry and other diagnostic methods should also be adopted. Patients need dynamic and comprehensive follow-up after ^131^I therapy.

CR was difficult to achieve in patients with higher sTg levels before initial ^131^I therapy. Our study also showed that CR was likely to be achieved if the sTg level was lower than 18.2 µg/l and that these patients might not need repeated therapy cycles. Wang et al (15) showed that the sTg level can be used to predict ^131^I treatment outcomes in patients with functional LNMs after PTC, and the optimal value of the cutoff point was 20.05 µg/l. Another report (16) also revealed that the sTg level (≤10.1 μg/l) was a key predictor of clinical outcomes in DTC patients. Lymph node size (5.5, 7.5 mm) is also a sensitive indicator for predicting clinical outcome in DTC patients with LNM (12, 15). Our study also confirmed that patients with smaller LNM size were likely to have excellent response after initial ^131^I therapy, especially for those <5mm. Furthermore, if we can predict the second PTS outcome, we may predict the patient’s treatment response. The current study found that patients with a negative second PTS were prone to have a good prognosis at the subsequent follow-up, and CR was achieved in 58/119 patients. Interestingly, our study showed that a positive PTS tended to be maintained in young patients with ^131^I+ LNM, but age was unrelated to prognosis. A higher age has been reported to be a negative factor for iodine accumulation in lung metastases from DTC (17). Although the sites of metastases are different, young patients may have a stronger iodine uptake capability. In clinical practice, we found that ^131^I+ LNM with light imaging was prone to turn negative and achieve good prognosis. However, Sa et al (18) considered that lesions with T/B_max_ (maximum target/background ratio) ≥8.1 will biochemically benefit from the next ^131^I therapy. It has been suggested that we should consider not only the ^131^I uptake capability of metastatic foci but also the number of tumor cells in lesions. It is possible that some ^131^I+ LNM only have a few thyroid carcinoma cells, and excellent outcomes can be achieved from ^131^I therapy.

Some limitations exist in our study. Only 13 patients were confirmed by pathology, and most LNMs were diagnosed by imaging and Tg/TgAb levels.

## Conclusion

In conclusion, repeated ^131^I therapy may be unnecessary in approximately one-quarter of patients, especially those with N0 or N1a stage disease, lower sTg levels, smaller LNM size, ≤2 residual/recurrent LNMs, negative ultrasound features and no ^131^I-LNM; furthermore, one-fifth of patients with contrary conditions may benefit from repeated therapy.

## Data availability statement

The original contributions presented in the study are included in the article/supplementary material. Further inquiries can be directed to the corresponding author.

## Ethics statement

The studies involving human participants were reviewed and approved by The Ethical Committee of Shanghai Tenth People’s Hospital (approval number: 22K256). Written informed consent to participate in this study was provided by the participants’ legal guardian/next of kin. Written informed consent was obtained from the individual(s) for the publication of any potentially identifiable images or data included in this article.

## Author contributions

All authors contributed to the study conception and design. Data collection were performed by YJ, SL, and XQ. Imaging analysis was performed by YH, XZ, and HC. The first draft of the manuscript was written by YJ and SL. The manuscript was reviewed by ZL and CM. All authors commented on previous versions of the manuscript. All authors contributed to the article and approved the submitted version.
